# Epidemiological evolution of repetitive strain injuries and work-related musculoskeletal disorders in Brazil: comparative analysis between 1990 and 2025

**DOI:** 10.47626/1679-4435-2026-1592

**Published:** 2026-09-27

**Authors:** Paulo Roberto Zétola, Caroline da Ros Montes D’Oca

**Affiliations:** 1 Universidade Federal do Paraná (UFPR), Curitiba, PR, Brazil

**Keywords:** cumulative trauma disorders, occupational health, chronic pain, occupational medicine

## Abstract

The objective of this study was to analyze the evolution of the epidemiological profile of repetitive strain injuries and work-related musculoskeletal disorders in Brazil, comparing the industrial and corporate scenario of the 1990s with national and regional data published between 2007 and 2025. A literature review with comparative analysis of secondary data was conducted, using as historical reference the analysis of 125 repetitive strain injuries cases among workers in the electro-electronic industry and, as a contemporary counterpart, studies based on data from Brazil’s Notifiable Diseases Information System, the Department of Information Technology of the Unified Health System, and recent literature. The historical sample showed a predominance of women (73.6%) and workers aged 31 to 35 years (28.0%), with initial emphasis on wrist tendinitis (49.6%) and shoulder bursitis (20.0%). At the end of follow-up, chronic pain syndrome was recorded in 65.6% of cases. Contemporary data showed a higher case concentration in older age groups, especially between 40 and 49 years in the national study from 2007 to 2021 and between 40 and 59 years in the 2018–2023 period. In Paraná, between 2014 and 2023, 5,694 cases were recorded, with female predominance (62.5%) and work shifts longer than six hours in 86.8% of notifications. The evolution of cases over time suggests greater clinical and occupational complexity of repetitive strain injuries and work-related musculoskeletal disorders, underscoring the need for cautious interpretation of indicators and integrated preventive strategies.

## INTRODUCTION

Repetitive strain injuries and work-related musculoskeletal disorders (RSIs/WMSDs) have historically constituted one of the main occupational health problems in Brazil, representing major causes of absenteeism, prolonged functional disability, and social security costs. Classified as multifactorial syndromes involving pain, functional limitation, and inflammation of the musculoskeletal system [^1^], these conditions have evolved in parallel with structural transformations in production and management models.

In the 1990s, Brazil experienced an “epidemic” of RSIs/WMSDs strongly associated with the industrial sector. The study by Zétola [^2^], involving 125 workers in the electronics industry, showed that rigid assembly lines, extreme repetitiveness, and the absence of breaks were the main determinants of illness. In that setting, the clinical case series consisted predominantly of young workers subjected to work rhythms dictated by machine pace [^2^].

With macroeconomic restructuring and the transition toward a service economy, the pattern of occupational illness underwent a qualitative shift. Although industry remains a relevant sector [^3^], the service sector, particularly banking, has become a critical area of concern. In this sector, pressure to meet productivity targets and intensive use of video display terminals are compounded by psychosocial factors that play a key role in the chronification of clinical conditions [^4^].

Recent data confirm the magnitude of this health problem in the current setting. Between 2018 and 2023, Brazil recorded 46,307 cases of RSIs/WMSDs, with the highest concentration among individuals aged 40 to 59 years [^5^]. Regional studies have also highlighted relevant occupational variables, such as prolonged working hours, breaks, and sick leave [^6^]. The emergence of telework and new forms of work organization in the post-pandemic period has introduced variables that still require monitoring, particularly with regard to the fragmentation of the workday and the ergonomic conditions of work outside the traditional workplace [^7^].

This context underscores the need to integrate the historical perspective of the 1990s with more recent indicators. The present study analyzes the epidemiological evolution of RSIs/WMSDs in Brazil, comparing the findings of Zétola [^2^] with data from 2007 to 2025 to identify the main changes in the epidemiological, clinical, and occupational profile of RSIs/WMSDs and their current challenges for Occupational Medicine.

### OBJECTIVE

The objective of this study was to analyze the epidemiological evolution of RSIs/WMSDs in Brazil by comparing the clinical, sociodemographic, and occupational profile observed among electronics industry workers in the 1990s with national and regional secondary data published between 2007 and 2025.

## METHODS

This is a descriptive epidemiological study using a quantitative and qualitative approach, conducted as a literature review with descriptive comparative analysis of secondary data. Its methodological structure was designed to enable a systematic comparison between the profile of RSIs/WMSDs observed in the 1990s and the national epidemiological landscape recorded between 2007 and 2025.

### DATA SOURCES AND BASELINE SELECTION

The primary source and historical reference point for this study is the dissertation by Paulo Zétola [^2^], which presents data on case distribution, demographic characteristics, and clinical diagnoses observed in a Fordist/Taylorist industrial setting at the end of the twentieth century. To update this epidemiological profile, cross-sectional studies and reviews published between 2013 and 2025 were selected, focusing on sources based on the Notifiable Diseases Information System (Sistema de Informação de Agravos de Notificação — SINAN) and medical records from Occupational Health Reference Centers (Centros de Referência em Saúde do Trabalhador — CEREST). The selected studies included national studies [^3^,^8^] and representative regional studies [^6^,^9^,^10^].

### ELIGIBILITY CRITERIA AND ANALYSIS

The inclusion criteria were:

- studies conducted entirely within Brazilian territory;
- presentation of consolidated epidemiological data on RSIs/WMSDs; and
- description of sociodemographic and occupational variables relevant to the occupational illness profile.

Strictly clinical studies focused on surgical, pharmacological, or therapeutic interventions that did not present a direct correlation with the profile of the economically active population were excluded.

Data extraction was conducted through structured reading of the selected files, with information categorized according to the data collection period, predominant industry, recorded diagnoses, and identified occupational outcomes.

### TEMPORAL COMPARISON STRATEGY

The temporal comparison was operationalized by contrasting key indicators: demographic profile (sex and age), diagnostic evolution (distinction between specific clinical diagnoses and ICD-10 diagnostic groupings), and predominant industry.

The heterogeneity of the sources led to limitations, however. While Zétola’s historical dataset [^2^] is based on clinical assessment and detailed diagnosis of individual cases, the contemporary studies analyzed in this review [^6^,^8^,^9^] rely predominantly on SINAN notifications, a system vulnerable to underreporting, variations in data completeness, and discrepancies in diagnostic classification.

Given the secondary nature of the sources, the variables analyzed were organized as qualitative and quantitative. Qualitative variables included sex, industry, clinical diagnosis or diagnostic grouping, sick leave, and occupational characteristics described in the studies. Quantitative variables included number of cases, proportions, percentages, predominant age group, incidence coefficients when available, and temporal variations. It should be stressed that incidence, prevalence, proportion of cases, and the absolute number of notifications represent distinct epidemiological measures; therefore, the indicators were used as descriptive markers of trends and epidemiological profiles, avoiding direct statistical comparison as though they were equivalent measures. The possibility of selection bias in studies involving specific occupational groups, such as bank workers, industrial workers, or populations receiving care in specialized services, is also acknowledged.

No hypothesis tests, meta-analysis, or calculation of aggregate measures across studies were performed due to the heterogeneity in methodological designs, populations analyzed, data collection periods, record systems, and diagnostic classification methods.

## RESULTS

The comparative analysis showed relevant changes in the profile of RSIs/WMSDs in Brazil in recent decades. It is important to note that the analysis did not try to estimate a single national prevalence, but rather to compare trends described in various sources, given their methodological limitations.

### SOCIODEMOGRAPHIC AND TEMPORAL PROFILE

In the 1990s, the affected population consisted predominantly of young workers, according to the electronics industry sample analyzed by Zétola [^2^]. The most frequent age group was 31 to 35 years (28.0%), followed by 26 to 30 years (20.0%) and 36 to 40 years (16.8%). Female predominance was also substantial, accounting for 73.6% of cases.

In contrast, contemporary data show a greater concentration in older age groups. In Pinto’s national study [^8^], covering the period from 2007 to 2021, 102,986 RSI/WMSD notifications were recorded in Brazil, with a slight predominance of females (52.1%) and the highest frequency among individuals aged 40 to 49 years (33.0%). In Gonçalves et al.’s nationwide study [^5^], covering the period from 2018 to 2023, 46,307 cases were recorded, with a higher proportion of males (53.13%) and a predominance of the 40-to-59-year age group (56.1%).

In the state of Paraná, Bueno et al. [^6^] identified 5,694 cases of RSIs/WMSDs between 2014 and 2023, with a predominance of women (62.5%) and self-declared White individuals (57.4%). The same study found that 86.8% of notifications involved workers with workdays of more than six hours per day and that approximately 37.5% of cases resulted in sick leave.

These data can be found in Table 1, which provides a descriptive comparison of the main indicators found by Zétola [^2^] and in the most recent national and regional studies.

**Table 1.** Descriptive comparison of epidemiological indicators of repetitive strain injuries and work-related musculoskeletal disorders

Variable	Zétola [^2^]	Pinto [^8^] / Brazil	Gonçalves et al. [^5^] / Brazil	Bueno et al. [^6^] / Paraná, Brazil
Period	1993–1998	2007–2021	2018–2023	2014–2023
No. of cases	125	102,986	46,307	5,694
Predominant sex	Female (73.6%)	Female (52.1%)	Male (53.13%)	Female (62.5%)
Predominant age group	31–35 years (28.0%)	40–49 years (33.0%)	40–59 years (56.1%)	35–49 years
Highlighted occupational variable	Electronics industry	Industry	National data/DATASUS	Working hours > 6 h (86.8%)
Methodological observation	Clinical case series	National SINAN	National DATASUS	State SINAN/DATASUS

Source: Prepared by the author.

DATASUS = Department of Information Technology of the Unified Health System; SINAN = Notifiable Diseases Information System (Sistema de Informação de Agravos de Notificação)

### DIAGNOSES AND EVOLUTION OF CHRONIC PAIN

There has been a transition both in diagnostic patterns and in the industries involved. In the historical setting, tenosynovitis accounted for 52% of the cases identified by Zétola [^2^], both at the initial stage and at the final stage of diagnosis and treatment. This diagnostic distribution was associated with wrist flexion-extension and ulnar-radial deviation movements resulting from the assembly of electronic circuit boards and soldering with small equipment [^2^].

The standout epidemiological aspect, however, was the triggering of chronic pain syndrome (CPS), present in 65.6% of cases at the final assessment, as detailed in Table 2.

**Table 2.** Main diagnoses of repetitive strain injuries and work-related musculoskeletal disorders

	Frequency	% of responses	% of cases
Chronic pain syndrome	82	40.80	65.6
Wrist tendinitis	61	30.35	48.8
Shoulder bursitis	24	11.94	19.2
Elbow epicondylitis	13	6.47	10.4
Carpal tunnel syndrome	9	4.48	7.2
Neck pain	5	2.49	4
De Quervain’s disease	4	1.99	3.2
Thoracic outlet syndrome	3	1.49	2.4
Total	201	100	160.8

In the final diagnosis, after clinical follow-up, CPS had the highest frequency, accounting for 65.6% of cases, followed by wrist tendinitis, at 48.8%, among 201 diagnoses identified. Because a single worker could have more than one diagnosis, the percentages should be interpreted as the proportion of occurrences within the case series, not as mutually exclusive categories or population prevalence estimates. This finding suggests that, although localized lesions remained relevant, persistent pain conditions also accounted for a substantial proportion of cases during clinical follow-up.

The severity of this condition is corroborated by studies identifying more severe pathologies. Azambuja et al. [^11^] identified complex regional pain syndrome (CRPS) and myofascial pain syndrome (MPS) in patients treated through the Brazilian Unified Health System (SUS), who had experienced pain for two years, on average. CRPS is characterized by pain disproportionate to the initial event, edema, and autonomic changes, indicating that pain chronification was associated with an unfavorable and difficult-to-manage clinical course [^11^].

Currently, diagnostic distribution reflects a greater complexity of illnesses that extends beyond isolated tissue injury. Although the M65 group (synovitis and tenosynovitis) remains frequent, representing the persistence of repetitive motor activity, a greater number of cases of dorsalgia (M54) and soft-tissue disorders (M79) have been reported in recent studies. This increase has been associated with inadequate furniture in the home environment during the COVID-19 pandemic and the lack of clear boundaries between work life and private life in remote work [^5^,^7^]. In this context, chronic pain transforms the body into a “repository of pain,” in which the meaning of having RSIs/WMSDs is inseparable from the loss of professional identity and social isolation [^12^].

However, the etiology of this chronification has undergone a fundamental qualitative shift: whereas in the industrial setting of the 1990s it derived predominantly from poorly healed physical injury, static exertion, and delayed diagnosis, in the current setting it shows a strong correlation with psychological distress arising from the new organization of work.

### PSYCHOSOCIAL FACTORS AND WORK ORGANIZATION

The analysis by Moronte & Albuquerque [^4^] finds that, in the banking sector, the contemporary management model, characterized by abusive performance targets, pervasive digital surveillance, and constant fear of unemployment, acts as a central trigger of occupational burnout. This psychological distress may manifest as musculoskeletal pain refractory to conventional physical treatment. Studies reviewed by the authors directly associate the use of anxiolytics and antidepressants among bank workers with the intense work pace and high psychoaffective burden, in which anxiety and depression act as amplifiers of pain perception. In this context, chronic pain is part of an illness process in which organizational mechanisms may encourage bodily overload in the face of fear and anxiety [^13^], and is often managed through self-medication [^1^], allowing workers to maintain their functional capacity despite symptoms.

### WORKING CONDITIONS AND OCCUPATIONAL OUTCOMES

Maintaining daily workdays longer than six hours remains a critical factor, accounting for 86.8% of documented cases in Paraná [^6^]. Another relevant finding concerns the increased use of self-medication with nonsteroidal anti-inflammatory drugs as a strategy for maintaining work productivity [^1^].

This phenomenon reflects the precariousness of contemporary working conditions and differs from the industrial setting of the 1990s, in which the literature focused primarily on biomechanics and task repetitiveness. The findings presented by Zétola [^2^] reflect trends identified in the studies analyzed in this review, without intending to estimate national prevalence, given the heterogeneity of the methodologies and regional samples. In the investigation of environmental risk factors for the development of cases detected by the company’s RSI/WMSD outpatient clinic, the most frequent factors were inadequate posture, repetitiveness, and work-system organization. Among the causal agents, greater frequencies were observed for lateral and medial wrist deviation, wrist flexion and extension, lack of task enrichment, and static exertion postures.

These working conditions, in a period marked by delayed diagnosis and less attention to occupational health problems, favored the progression of musculoskeletal injuries, aggravated by lack of knowledge about the disease, delayed diagnosis, and lower availability of preventive strategies and clinical follow-up at the time. This situation changed considerably over the years with advances in knowledge about the disease and its progression, as can be observed in the contemporary studies included in this literature review.

This evolution in knowledge made it possible to recognize that the implementation of prevention and health surveillance programs not only reduces case severity but may also change the epidemiological understanding of the condition. As demonstrated by Zétola [^2^], the transition to an early-diagnosis model revealed that timely intervention can significantly reduce the duration of sick leave but also highlights the persistence of chronic conditions that previously went unnoticed or were incorrectly classified. Thus, technical improvements in the biomechanical conditions of work (physical ergonomics) have come to coexist with an increased burden of diseases related to psychosocial factors. Refinement of early diagnostic methods has shown that, even with reductions in classical physical risks, organizational pressure and occupational stress may still contribute to the emergence of new manifestations of RSIs/WMSDs, now intrinsically related to psychological distress and mental exhaustion.

As an interpretive complement, studies of bank workers were considered solely as a sector-specific example of the influence of work organization, pressure to meet targets, task intensification, and psychosocial factors on occupational illness. This focus helps illustrate the transition from the classic profile of RSIs/WMSDs, strongly associated with industrial repetitiveness, to contemporary forms in which biomechanical, organizational, and psychosocial factors coexist.

However, findings concerning the banking sector should not be interpreted as representative of RSIs/WMSDs as a whole in Brazil, as they derive from a specific occupational category and from studies with different objectives, designs, and populations. Thus, Table 3 represents an illustrative synthesis of a service sector rather than a national comparative basis equivalent to epidemiological studies based on SINAN or DATASUS data.

**Table 3.** Sector-specific example: studies on work organization, psychosocial factors, and illness among bank workers

	Study	Title	Main results
4	Geremias et al., 2017 [^14^]	Prevalência do diabetes mellitus associado ao estresse ocupacional em trabalhadores bancários, Minas Gerais, Brasil	Reports working conditions in banks as sources of tension: excessive working hours, intensification of activities, pressure for results, high performance demands, unpredictability of situations, and dismissals related to failure to meet targets.
5	Ferreira & Carvalho, 2017 [^15^]	Saúde do Trabalhador: Avaliação da Qualidade de Vida no Trabalho de Bancários	Indicates that banking work has undergone a transformation, with workers now selling products and being subjected to work-control techniques, particularly productivity target-monitoring systems.
6	Ziliotto & Jaques, 2017 [^16^]	As contingências do trabalho bancário: um estudo sobre os mecanismos de mediação e defesa do sofrimento	Relates aspects of banking work to negative impacts on workers’ health: headcount reductions, technological development and modernization of work processes, increased demands for professional training, and conditions of wage and contractual insecurity in the sector.
7	Silva & Navarro, 2012 [^17^]	Organização do trabalho e saúde de trabalhadores bancários	Relates productive restructuring to increased productivity and profitability in the sector, as well as reductions in job positions, intensification of the work pace, task overload, and increased pressure and control over bank workers, with impacts on workers’ health.
8	Medeiros et al., 2017 [^18^]	Sequestros de bancários e seus impactos psicossociais e na saúde do trabalhador	Considers that changes in the capital-labor relationship resulting from the application of neoliberal ideology in the banking sector generated precariousness in labor relations (lower wages, increased outsourcing, and temporary employment contracts), an increased work pace (related to targets and deadlines), and a high psychoaffective burden (related to demands for perfect performance and constant threats regarding career prospects).
9	Silva et al., 2008 [^19^]	Perfil do absenteísmo em um banco estatal em Minas Gerais: análise no período de 1998 a 2003	Finds a change in the pattern of illness and sick leave, with a reduction in cases of musculoskeletal disorders and an increase in mental disorders.
10	Picheth & Ichikawa, 2015 [^20^]	Uso de ansiolíticos e antidepressivos por bancários: um estudo de representações sociais	Relates physical and mental suffering and illness to the organization of work in the banking sector (high levels of pressure for productivity).
11	Petarli et al., 2015 [^21^]	Autoavaliação do estado de saúde e fatores associados: um estudo em trabalhadores bancários	Attributes a direct impact on the health of bank workers to changes in the organization and management of work in the sector: mass layoffs, automation, outsourcing, and business reengineering, with reductions in hierarchical levels, precarious working conditions, and multifunctional tasks.
12	Murofuse & Marziale, 2001 [^22^]	Mudanças no trabalho e na vida de bancários portadores de Lesões por Esforços Repetitivos: LER.	Relates musculoskeletal disorders to changes in working conditions.

Source: Prepared by the author.

In the historical database analyzed by Zétola [^2^], the implementation of a structured prevention and rehabilitation program was associated with improvements in indicators of severity and return to work. In the comparison between the 1995–1996 and 1997–1998 periods, the number of RSI cases decreased from 75 to 42, with a proportional decrease from 3.62% to 2.15% relative to the mean number of workers. Over the same period, the mean duration of sick leave decreased from 34.48 to 12.71 days.

These findings suggest that strategies involving early diagnosis, clinical surveillance, ergonomic adjustment, and functional rehabilitation may contribute to reducing the severity of new cases and promoting return to work, although they do not eliminate the possibility of chronic progression in some affected workers.

Recent changes in work, including telework, longer working hours, and the fragmentation of collective routines, should also be taken into account in the contemporary analysis of RSIs/WMSDs. However, the studies analyzed in this review do not allow the epidemiological evolution of RSIs/WMSDs to be attributed solely to these modalities. These factors should be interpreted together with biomechanical exposures, work organization, breaks, working hours, and limitations of notification systems.

## DISCUSSION

The comparative analysis of Zétola’s findings [^2^] and the contemporary scientific literature suggests a change in the epidemiological profile of RSI/WMSDs in Brazil, from a scenario initially described among workers in the electronics industry to a more heterogeneous perspective involving more diverse sectors, older age groups, and greater diversity of diagnoses and occupational outcomes. This transition, however, should be interpreted descriptively, considering that the sources analyzed differ in methodological designs, populations, periods, and recording methods.

### THE SUBJECTIVITY OF PAIN AND THE CHRONIFICATION PROCESS

The frequency of CPS observed by Zétola [^2^] in 65.6% of cases at the end of follow-up underscores the relevance of clinical chronification in RSIs/WMSDs. This finding should not be interpreted as population prevalence, but rather as the proportion observed in an occupational clinical case series. Nevertheless, it helps demonstrate that some cases may progress from localized lesions to persistent conditions of greater diagnostic and functional complexity.

Subsequent studies also point to this complexity. Azambuja et al. [^11^] identified cases of MPS and CRPS in patients treated at an occupational health outpatient clinic, demonstrating that persistent pain may be associated with poorer functional outcomes. These findings highlight the need for early diagnosis, clinical follow-up, and functional rehabilitation, without allowing direct inference regarding the population-level magnitude of these conditions.

### PSYCHOSOCIAL FACTORS AND WORK ORGANIZATION

In addition to classical biomechanical factors, contemporary literature highlights the role of organizational and psychosocial aspects in the current profile of RSIs/WMSDs, including pressure for productivity, performance control, reduced breaks, job insecurity, and reduced autonomy. The banking sector is a relevant example of this process, particularly because of the combination of targets, work intensification, and intensive use of technologies [^4^]. However, these findings should be interpreted as complementary sector-specific evidence rather than as representative of RSIs/WMSDs as a whole in Brazil.

### REMOTE WORK, TELEWORK, AND NEW TRENDS

The transition to remote work and the expansion of new forms of work organization have introduced variables that still require monitoring, such as improvised workstations, fragmentation of the workday, and reduced collective breaks. However, the studies analyzed in this review do not allow the epidemiological evolution of RSIs/WMSDs to be attributed solely to these modalities. These factors should be interpreted together with biomechanical exposures, work organization, breaks, working hours, and limitations of notification systems.

### THE “CONCEALMENT” PHENOMENON AND LIMITATIONS OF INFORMATION SYSTEMS

Self-medication and underreporting remain relevant limitations in understanding the magnitude of RSIs/WMSDs. In addition, the shift from individualized clinical assessments to aggregated notification databases, such as SINAN, may diminish diagnostic specificity and hinder direct comparison between studies, particularly when chronic conditions and complex pain syndromes are analyzed.

## CONCLUSIONS

The comparative analysis indicates that RSIs/WMSDs in Brazil have undergone relevant changes in their epidemiological profile, without this representing the disappearance of the classical industrial pattern. In Zétola’s historical sample [^2^], women predominated (73.6%), the 31–35-year age group was the most frequent (28.0%), and localized upper-limb lesions stood out, although CPS accounted for 65.6% of cases at the end of follow-up.

A greater concentration in older age groups was observed in contemporary studies, along with the continued importance of the industrial sector as a major component of notifications and greater emphasis on organizational variables. Among industrial workers, 17,537 notifications were identified between 2007 and 2013, corresponding to 47.9% of national notifications during the period. In Paraná, between 2014 and 2023, 86.8% of cases occurred among individuals working more than six hours per day, and approximately 37.5% resulted in sick leave.

These findings underscore the fact that the prevention of RSIs/WMSDs should integrate ergonomic measures, clinical surveillance, work organization, adequate breaks, early diagnosis, and functional rehabilitation. Given the secondary nature of the data and the heterogeneity of the sources, the conclusions should be interpreted descriptively, avoiding direct equivalence between incidence, prevalence, clinical proportions, and the absolute numbers of notifications.
